# Organizing as negotiation: the construction of a pathway in Norwegian mental health services

**DOI:** 10.1186/s13033-021-00451-5

**Published:** 2021-03-19

**Authors:** Tine Nesboe Toerseth

**Affiliations:** 1 The Mohn Centre for Innovation and Regional Development, Western Norway University of Applied Sciences, is a Research and Competence Centre within the Field of Responsible Innovation, Bergen, Norway; 2 The university of Bergen, Bergen, Norway

**Keywords:** Clinical pathway, Mental health services, Standardization, Autonomy, Discretion, Health profession, Institutional logics

## Abstract

**Background:**

In 2015, a decision was made to implement clinical pathways in Norwegian mental health services. The idea was to construct pathways similar to those used in cancer treatment. These pathways are based on diagnosis and evidence-based medicine and have strict timeframes for the different procedures. The purpose of this article is to provide a thorough examination of the formulation of the pathway “mental illness, adults” in Norwegian mental health services. In recent decades, much research has examined the implementations and outcomes of different mental health sector reforms and services in Western societies. However, there has been a lack of research on the process and creation of these reforms and/or services, particularly how they emerge as constructs in the contexts of policy, profession and practice.

**Methods:**

A qualitative single case study design was employed. A text and document analysis was performed in which 52 articles and opinion pieces, 30 public hearing responses and 8 political documents and texts were analysed to identify the main actors in the discourse of mental health services and to enable a replication of their affiliated institutional logics and their views concerning the clinical pathway. Additionally, ten qualitative interviews were performed with members of the work group responsible for designating the pathway “mental illness, adults”.

**Results:**

This article shows how the two main actor groups, “Mental health professionals” and “Politicians”, are guided by values associated with a specific logic when understanding the concept of a clinical pathway (CP). The findings show that actors within the political field believe in control and efficiency, in contrast to actors in mental health services, who are guided by values of discretion and autonomy. This leads to a debate on the concept of CPs and mental health services. The discussion becomes polarized between concern for patients and concern for efficiency. The making of the pathway is led by the Directorate of Health, with health professionals operating in the political domain and who have knowledge of the values of both logics, which were taken into consideration when formulating the pathways, and explains how the pathway became a complex negotiation process between the two logics and where actors on both sides were able to retain their core values. Ultimately, the number of pathways was reduced from 22 to 9. The final “Pathway for mental illness, adults” was a general pathway involving several groups of patients. The pathway explains the process from diagnosis through treatment and finalizing treatment. The different steps involve time frames that need to be coded, requiring more rigid administrative work for compliance, but without stating specific diagnostic tools or preferred treatment strategies.

**Conclusions:**

This article shows that there is also a downside of having sense making guided by strong values associated with a specific institutional logic when constructing new, and hopefully better, mental health care services. This article demonstrates how retaining values sometimes becomes more crucial than engaging in constructive debates about how to solve issues of importance within the field of mental health care.

## Background

Most Western countries are struggling with the rising cost of health care services. There is a common view that better organizing these services is the answer to the issues of lack of resources and increased demands [1]. This context reinforces an ideology of increased monitoring and transparency, where management is given more power to ensure that hospitals are better controlled and more predictable [2–4]. These elements all lead to standards and standardization being proposed as solutions [5, 6]. Organizing health care services through standardized clinical pathways (CPs) occurs in several areas of Norwegian health care, with the implementation of CPs within cancer treatment as the largest national introduction of standardized service production [7]. The European Pathway Association (EPA) defines the standardization of care processes into CPs as “a methodology for the mutual decision making and organization of care for a well-defined group of patients during a well-defined period” [8]. The method defines goals and decision making on which measures to include in the treatment. The measures should reflect evidence, best practice solutions, and the involvement of the patient [9, 10].

More than once, politicians have been accused of not prioritizing mental health, leading to waiting lists as well as an eminent capacity and resource problem.^1^ Furthermore, over recent decades, Norwegian mental health services have met with much criticism from professionals and patients within the field [11]. This has led to a debate regarding the organization of mental health care. Often polarized viewpoints circulate around terms of efficiency and/or care, user participation and/or medicalization [12, 13]. This battle regarding the organization of health care services is often presented in the literature as disputing logics that influence health care practices in different ways. These logics contain a particular set of behaviours, rules and norms and function as guiding principles for the actors inhabiting them [14–18]. In January 2016, the Ministry of Health and Care Services officially assigned the production of several CPs in mental health services to the Directorate of Health [19] CPs in somatic medicine were imported from Danish health care, raising a desire to copy pathways in mental health services from Denmark as well. The CPs in Danish mental health services had an outlook on diagnosis with strict time frames and different standardized manuals to follow, thus influencing discretion and autonomy of individual professionals [7].

The analysis provided in this article builds on the different views of the main groups of actors and interpretations of CPs within the field of mental health services. Understanding the logics and its affiliated values is vital when analysing the process that led to the final product [20]. Elaborating this issue further leads me to the following research question.

*How do actors in the field of Norwegian mental health services interpret and understand the concept of CPs, and in what ways did this affect the construction of a pathway?* This article starts by elaborating the main actors in the field of mental health services before discussing the current elements of what constitutes a preferred way of organizing health care services today. Professionalization and its discretional activities contrast with scientific bureaucratic medicine. In enabling an explanation of the different actors’ understanding of CPs, I present theory on institutional logics to show how different values associated with a logic influence the actors’ sense making and interpretation of a CP. In the methodological section, I show how a case study in combination with discourse analysis enables me to categorize my textual analysis into two main institutional logics. The analytical part explains how the pathway became a complex negotiation process between the two logics and where actors on both sides were able to retain their core values. Finally, this paper concludes that deliberative policy making has a pitfall when the agents responsible for the construction is guided by values belonging to different institutional logics, because withholding these causes polarization of the debate, potentially influencing the final product negatively.

This paper’s contribution, is twofold. By examining the development of a new policy, I offer a supplementary approach for those studying health organization and implementation [21]. As Dobson [22] highlights, the unconscious use of linguistics by the enactors of policies becomes a reflection of their social worlds. By elaborating this concept, I wish to demonstrate that the different values of different actors influence the implementation of policy development. Furthermore, I extend the literature on CPs by researching issues other than their use in an individual care setting as well as broadening an understanding of institutional logics’ empirical expression. Johansson and Waldorf [23] point to the lack of studies on how actors use multiple sets of expectations to cope with an environment at the intersection of several institutional fields. Conclusively, they encourage researchers utilizing institutional logics to “know much more about the informal organization, the chaos and the ‘muddling through’ [24], in decision-making processes, and the actors’ tiring negotiations and power struggles”. This article aims to answer these calls.

### The field of mental health services in Norway

Mental health services as a field encompasses many actors, and the field encounters ongoing criticism from different perspectives [11, 25–31], Norwegian mental health services is no exception. As the field of mental health services with its actors does not anticipate shared meaning [25–31], this paper utilizes a more practical definition by DiMaggio and Powell [32] that suggests that a field is “*those organizations that, in the aggregate, constitute a recognized area of institutional life: key suppliers, resource and product consumers, regulatory agencies, and other organizations that produce services or products*”. Furthermore, they concede that the struggles to write the rules and control the resources are all a part of the construction of an organizational field [33]. Finally, fields become centres of debate in which competing interests negotiate issue interpretation [34]*.*

In Norway, mental health services are a part of the welfare state that aims to provide care and help to inhabitants in need of it. The key terms in the welfare reforms of 1980 were “normalization” and “autonomy”, leading to a deinstitutionalization of mental health  services, meaning that people suffering from mental disorders received health services where they lived. These ideas were collected mainly from user movement groups reflecting ideologies of recovery [9]. The concept of recovery is debated, but overall recovery can be viewed as a phenomenon including social processes and everyday practices in mental health care. The focus is on society, living conditions and social processes. Home, work and activity as well as education, money, friends and community all play a role in the recovery process [35, 36].

These tendencies brought forward “The escalation plan for mental health care”, a large-scale political reform from 1998 to 2008 built upon White paper no. 25, “Openness and wholeness: a report on mental health care and services”. The reform and its overall goals were described in government proposition no. 63 (1997–1998) [37], and the reform aimed at quantitative and qualitative improvement of the services and was built upon values emphasizing independence, autonomy and the ability to master one’s own life. Furthermore, sectors and service providers were encouraged to establish networks across sectors and administration levels.

This focus on recovery was further emphasized in the establishment of drug-free services based on requirements from "The Joint Action for Drug-Free Services," I 2011, which is an association of the organizations National Association for Relatives in Mental Health (LPP), Aurora Support Association, Mental Health, White Eagle, and We Shall Overcome (WSO) [38]. In 2015, a letter from the Minister of Health was sent to each regional health enterprise demanding the establishment of drug-free mental health care services by 1 June, 2016 [39] thus providing patients with an increased ability to influence their own treatment The aim was to further empower patients in the field and reduce the use of coercive measures.

Despite the focus on recovery and users, Ekeland [40], in his review on Norwegian mental health services, shows that despite the action plan [37] in which the government tried to involve the user perspective [37, 40], there exists a hegemonic position within mental health services that leans towards medicalization and a bio-medical model as well as increased psychologization, with the cause of the problem being placed within the individual instead of examining structural issues like social support [30, 40].

Furthermore, numerous reports have found weaknesses regarding challenges in the organization and execution of treatment within Norwegian mental health care [41–44]. A common conclusion from these studies is a lack of equal services, standardization and quality of different service providers in different parts of Norway.

The field of mental health services examined in this paper circulates around three different groups of actors: 1. politicians deciding and executing mental health policy, 2. health professionals and patients operating in the field, and 3. user and interest groups aiming to improve different psychiatric services. Moreover, within the field, we find both organizations and individuals who inhibit the prospect of expressing logics, values and perspectives that potentially influence patients and organizations as well as the field in general. Furthermore, different health professions base their logics on what psychiatric illnesses are and how to treat them from many angles, ranging from dedication among doctors believing in the use of medication to improve an unbalanced brain to social workers believing in peer support and care, representing the other side of the spectrum.

### Professionalization in mental health care: discretion and autonomy as core values

Professionalization in health care is often referred to as discretion practised autonomously by an individual practitioner or professional group [45]. Professional actors do not follow their own selfish interest, as their profession is developed to solve problems and/or issues for the better of society. Therefore, their ethics is based on the needs of the client [46], and professional groups define performance standards as well as ethical codes for their members in accordance with thorough training [47–49]. The “power” of a profession includes the identification and safeguarding of the content and practices of its work [45, 49]. Furthermore, Freidson [50] concludes that autonomy and discretion are more important than professional knowledge and expertise because upholding autonomy is the only way a profession can secure control and protect its standards, autonomy and discretion [45].

Further, he [50] argues that professionalism is an ideal type of organization of work (or what he terms “a third logic”), where health professionals act as mediators presiding over the interests of the state by serving the needs of the public and demands of patients [51]. The arguments above all rest on the idea that professional knowledge should be valued in such a manner that health professionals have the freedom to execute their work without further external restrictions [51].

Although health professionals within the field of mental health services believe in their own discretional evaluations and behaviour, few studies have proven their abilities. A pioneer in this field was Meehl, who in 1954 wrote the book Clinical versus statistical prediction, in which he compared clinicians’ discretional activities and simplified mathematical formulas. His conclusions clearly indicated that experts’ evaluations were poorer than even the simplest mathematical model. The same conclusions were enhanced in 1998 by Garbs in “Studying the clinician”. Here, he performed a thorough examination of research on the connection between the experience and quality of clinical discretion within the field of behavioural analysis, psychological diagnosis and evaluation of personality and psychopathology. Since then, hundreds of studies have been performed that compare professionals’ discretion and statistical, linear models and reliable outcomes; however, the correlations are weak and/or non-existent on the discretional side [52, 53]. This lack of linear significance has been explored by many researchers, including Hoghart [54], Kahneman [55], and Kirkeboen [53]. A common understanding of the phenomenon is the lack of evidence-based frameworks for understanding individual behaviour as well as different biases in cognitive interpretations of the world [53].

However, despite the evidence against relying too heavily on discretion, health professionals within this field believe in their professional abilities to make correct evaluations and judgements. There could be many reasons for this, such as threats against one’s professional self, economic reasons and common myths about professionals’ discretional abilities [53]. Furthermore, this article shows that one such explanation is withholding values belonging to an agent’s professional identity.

### Scientific bureaucratic medicine

Scientific bureaucratic medicine is a term from Harrison and Ahmad’s [56] research on care pathways and its following guidelines. It is called “scientific” in the sense that it draws on the accumulated evidence of large-scale research and “bureaucratic” in the sense that it translates the output of such research into a particular species of bureaucratic rule for application in medical care organizations [56]. The concept could be understood scientifically in light of evidence-based medicine (EBM) and bureaucratically in light of new public management (NPM).

EBM is grounded in best practice solutions, guidelines, protocols, and checklists for standardizing procedures in the belief that it is the best way to reduce unwanted variation in diagnosis and treatment [57, 58]. In the Norwegian context, EBM was introduced in 1995 and institutionalized in 2004 through the establishment of the Norwegian Knowledge Centre for Health Services [59].

EBM has found an ally in NPM, a concept motivated by increased efficiency as the desired outcome and inspiring public reforms across the Western world [2]. The focal point is adopting market-based models aiming at a broad focus on performance measurements and control measures within the public sector, to be monitored at the political level [2]. Within health care, NPM has been an international trend during the last three decades [60–63], and the implementation of performance-based financing in Norwegian somatic hospitals in 1997 and within mental health services in 2017 were two of several NPM ideas within health care [5, 60–64]. However, despite the influence of NPM and EBM in public health care, there are huge differences in understandings and opinions of these concepts, placing them as conflicts between core opposing values such as care and quality treatment versus financial objectives [64, 65] and, furthermore, between professional and political work [66, 67].

### Institutional logics

The foundational work on institutional logics is viewed as “organizing principles” [68]. Fundamental to this perspective is the belief that the interests, identities, values, and assumptions of individuals and organizations are embedded within prevailing institutional logics [69]. Thornton and Ocasio [70] define institutional logics as.

“*the socially constructed, historical patterns of material practices, assumptions, values, beliefs, and rules by which individuals produce and reproduce their material subsistence, organize time and space, and provide meaning to their social reality”.*

Despite the fact that an institutional logic consists of several elements that the actors utilize when making sense of the world, there is an understanding that this sense making consisting of assumptions, beliefs, rules and material practices is based on values. This makes.

“…*value central to an institutional logic: a presumed product of its prescribed practices, the foundation stone of its ontology, the source of legitimacy of its rules, a basis of individual identification, a ground for agency, and the foundation upon which its powers are constituted”* [71].

Institutional logics influence actors’ sense making when they identify with the collective identities of an organization and/or profession [69, 72, 73]. Within professional fields, professional logics offer the identities through which professionals make sense of who they are “Professional role identity is enabled and constrained by the institutional environment and provides interpretations that professionals adopt” [74]. The relationship between institutional logics and identity is recursive—each shapes the other, institutional logics give identity to those who share them, and those who share identity mutually reinforce their shared logics. Identity provides the link between the field-level meaning, institutional orders, and the sense making of individual human actors [68, 69, 75–77]. In and between different situations encountered by actors, they activate a variety of social identities based on different institutional logics [78]. Johanssen and Waldorff [23] examine how research within this domain of institutional logics has had a tendency in the empirical expression of logics to lack a common ground for operationalization, see e.g. [79, 80]. Studying how actors in the field of mental health services engage in a negotiation process can provide empirical insight into how an operationalization based on Thornton and Ocasio’s [70] initial definition expresses itself.

## Methods

### Data sources

To understand the making of a pathway in its context and how the different actors make sense of the phenomenon, a methodological outlook through a case study is fruitful [81].

### Text and documents

First, the written material in the public realm of CPs in mental health services is analysed. This process involves examining chronicles, political speeches, documents and hearing responses as well as the pathway. A more specific overview of the texts and documents can be found in Table 1, Data sources.This part of the analysis focuses mainly on identifying institutional logics.

**Table 1:** Data sources

	Articles and opinion pieces (Aug. 2015–Nov. 2017)	Public hearing responses to clinical pathway	Political documents and texts	Interviews affiliated with “the work group”
The government	8		5	
Health professionals	33	15		Psychiatrists: 2 Psychologists: 2 Psychiatric nurses: 2
User groups & special interest organizations	11	15		2
Directorate of Health			3	3

### Qualitative interview

The interview data come from ten in-depth interviews with members of the work group designated by the Directorate of Health to compose the “pathway for mental illness, adults”.

### Selection and recruitment

The informants were found via the Directorate of Health web page. The interviewees were strategically selected based on Creswell & Creswell 2018s criterion of optimal variation [82] so that actors from different professions as well as the perspectives of patients and user groups were included. To control for variations in personal opinions [83], interviews were carried out with two representatives from equal backgrounds where possible.

### The execution of the interviews

The interviews were collected between August and October 2018, took place either over Skype or face to face, and lasted between 40 and 60 min. The informants were asked about their own ideas of a CP, what they thought about it initially and the result. In addition, I asked them about the process of making the pathways, such as differences of opinions and whether there were any power imbalances in the group. Furthermore, they were asked to provide a brief account of what they considered the greatest challenges within mental health care and to what extent the CPs improved these elements. All the interviews were taped and transcribed.

### Ethical issues

Furthermore, approval for the project was provided by the Norwegian Centre for Research Data (NSD). The gathering of data followed the ethical guidelines of the NSD, including obtaining written informed consent for my interviews and explaining the purpose of the study. The documents were sent by e-mail before each interview.

## Analytical strategy

### Discourse analysis

A discursive approach is a choice when one wants to perform an in-depth, methodical analysis of a specific phenomenon. The term discourse covers the basic idea that language is structured in different patterns when we interact within different social domains [84, 85]. Discourse analysis is not just one approach but also a series of interdisciplinary approaches that can be used to explore many different social domains in many different types of studies [85]. When linking a certain discourse with a certain expert community, it is not simply a question of a particular group of experts having a common set of goals and language; it is what the experts want and know how to impose on the audience [86].

### Identification of the institutional logics

To understand a field’s belief system and practices is a complex process, I follow the examples of Reay and Hinings [17] and Scott et al. [14] by examining indicators that identify the different actors’ logics, meaning to look for similarities in the expression within the already established elements. These are material practices, assumptions, values and beliefs based on Thornton and Ocasio’s [70] definition of institutional logics and how they unfold in the context of the the idea of a CP.

The operationalization of the logics consists of elements that enable a structured coding of the written material. NVivo (qualitative data analysis software) enabled me to categorize my material in a structured manner. Later, I reread the material and looked for patterns that enabled replication. In this part of the analysis, I was able to identify three main actors. However, it was clear that the overall and generalized values, assumptions and beliefs about a CP were shared by health professionals working within mental health care as well as user groups. Therefore, during the analytical part, the user/patient perspective is merged into one, enabling a comprehensible reproduction of the textual analysis. A complete overview of this analysis is shown in Table 2. *“Clinical pathway and institutional logics”.*

**Table 2:** Clinical pathway and institutional logics

Characteristic	Mental health professional/ patient logic	Political logic
Material Practice	EBM & standardization interfering with discretion, making it hard to provide correct patient treatment	CP secures correct and best practice execution of services
Assumption	CP is unsuitable for Mental health care services because each patient needs individual care	CP is the solution to capacity problems, unwanted variation, and inefficient treatment
Values	CP collides with discretion and autonomy	CP secures control, effiency and quality
Beliefs	CP is only concerned with efficiency and cost reduction, making patient care and recovery harder	CP will improve the services
Rules	CP opposes professional values: Humanity (patients), care (services), knowledge and autonomy	CP requires rules and standards to be monitored and controlled

## Results

This part of the paper seeks to provide a thorough examination of the making of the pathway in Norwegian mental health services. The Directorate of Health established an external work group in 2016, aiming to finishing a process and evaluation plan to be delivered to the Ministry of Health and Care Services by 1 April the same year. Shortly after, work groups for each pathway were established. Each work group consisted of professionals in the field as well as patients and their affiliated organizations and unions. During the process, different conferences were arranged where agents provided expertise and relevant actors were free to state their opinion. In addition, the pathways were sent out for public hearings [19]. Originally, the pathways were intended to be implemented in September 2018; however, delays brought them to life on 1 January 2019 [19, 87]. After the prime minister, Solberg, announced the reorganization of mental health care services into CPs in 2015 [88], a tense media debate regarding mental health treatment and service organization occurred. This media debate is followed in the first part of the analysis, where the main goal is to identify each group’s institutional logics and the values affiliated with them. The second part of the analysis examines the process as well as the final result.

### The media debate

Shortly after the prime minister announced the plan to implement CPs in Norwegian mental health services in 2015 [88], a tense media debate arose [19, 87, 89].

The debate focused for the most part on the negative effects standardization potentially has on individual care and treatment. A common view was an expectation that the pathways would be copied from Danish health care and somatic cancer treatment, leaving out much of the discretion and autonomy of each individual provider, elements that were characteristic of treatment facilities when the idea was launched. The criticism from health professionals was met by politicians with a promise to listen to both professionals and patients but without changing their ideas about implementation. The debate, however, shed light on the different groups’ institutional logics, and a more detailed analysis follows.

### The political logic: the CP as the solution to issues in mental health services

Recent years have revealed issues of capacity within mental health care, and in accordance with NPM- and EBM-inspired beliefs within the political logic, increased control and standardized measures could be solutions to some of these issues. The wish to implement CP in mental health services was hailed as an approach that could improve these services and the issues they face when Prime Minister Solberg first elaborated the idea in 2015 [90]:

“*We will make a radical grip to make diagnosing and treatment of mentally ill patients faster, better and more predictable. We will introduce clinical pathways into mental health services*”.

There is a firm belief that this way of organizing health care services leads to more efficient services. Standardization is the preferred strategy for achieving at this goal. This is further explained by Minister of Health Høie [91] when he states*:*

*“The methodology behind clinical pathways is about standardizing the patient’s services with two main objectives: to reduce unnecessary waiting time and to secure that everyone gets the best possible treatment*”.

Furthermore, CPs combine EBM with NPM, making the concept belong to the idea of scientific bureaucratic medicine. This form of medical logic is based on and promotes the values found within political logics, namely, efficiency, quality, and control.

### Political logics values of efficiency, quality and control

These values function as cornerstones in several issues regarding governing public health care, and in relation to mental health services, where these issues have been frequently discussed, there is an almost taken-for-granted assumption that control and standardization, namely, through CPs, are the solutions. This comment from Prime Minister Solberg [92] emphasizes this assumption:

“*Clinical pathways in mental health services would lead to less discrimination by implementing standards for the content in the examination of and treatment strategies for the patient as well as more predictability for the patient with timeframes for the different steps”.*

Control, efficiency, and quality guide arguments on how and why CPs are the best way to organize mental health services. The way to control the services is by outsourcing responsibility that can be monitored and thereby controlled by the political level. This will hopefully lead to better quality and efficiency, as is stated explicitly by Minister of Health, Høie [93]:

*“Clinical pathways will not only provide patients with more predictability but will also give practitioners in the different parts of the services more predictability. They will clarify what responsibility the different practitioners have during examination and treatment”.*

There is a conspicuous absence of a softer language associated with work in this field. Compassion, trust, and care are all important in regard to understanding work within a mental health institution and are often utilized through discretion or autonomy. However, these elements are more difficult to quantify and standardize and are thus much left out of the discussion on CP at the political level. Conclusively, the different beliefs, assumptions and material practices found in this institutional logic come from the core values of efficiency, quality and control as the drivers of the CP.

### Health professional logic and the conflict between standardization and individual care

The overall assumption within this logic is that CPs are unsuitable because each patient needs individual care, making standardized practices unsuitable for patients within the field of mental health. Individuality is closely linked to discretion and what psychiatrist Aare and Mehdi [94] pinpoint in their chronicle *The house of cards that collapses in mental health services:*

“It’s about time to fight for the patient’s right for individuality and professionals calling to be professional”.

This individuality is further emphasized in the overall debate as something that characterizes patient treatment within the field, and there is consensus that individuality, and not equality, is something that characterizes good patient treatment. The way the CP unfolds from the outlook of health professionals is portrayed as something generally negative and what Doctors Vogt and Pahle [95] state in their chronicle*: “Equality on assembly line”:*

*“The government wants to standardize mental health care in clinical pathways and sells it as equal treatment. The basic idea of what it means to help is at risk. Clinical pathways belong more to Toyota than humane mental health services…”.*

The rationale behind CPs is believed to be the same as that behind NPM, efficiency and cost reduction. This brings forward an assumption of concern with either efficiency and cost reduction (political level) or patient and care (health professional level), leading the debate into polarization. The polarization originates from professional beliefs in discretion and autonomy as the ideal way to practise mental health care.

### Values: the CP interferes with discretion and autonomy

The number-one guiding value in a health professional logic is discretion, closely followed by autonomy. An overall understanding of the public debate made visible that withholding these values in the making of the pathway was of vital importance. The consequences of losing their discretion are addressed by psychiatrist Aare and Mehdi [94];

*“The values that form the basis for the patient’s health service are not compatible with clinical pathways. In the worst case, they are making new rules on how patients and practitioners should organize themselves. Rules that take away their freedom and creativity”.*

The fear of losing their freedom in terms of executing treatment and providing care is in accordance with Freidson [50], who elaborates how health professionals secure control and determine their standards by protecting autonomy and discretion [50].

*“What are the core values behind clinical pathways? Control! Control over professionals, and a system one experiences as uncontrollable, cost reduction and efficiency, efficiency, efficiency!”*

The above quotation from the two doctors Pahle and Vogt [95] further enhances the protection of boundaries by discrediting the opponent’s values as being unconcerned with patients. The polarization of the debate is, namely, done by agents of the health professional logic, and the arguments are centred around how a focus on efficiency means being concerned not about patients but about cost reduction. Health professionals view standardization as incompatible with individual adaptation and flexibility, a major part of their work practice.

### When standardization meets individualization, the user perspective meets political values

Anne Grethe Teien, a former patient, responds to the post from Tove Gundersen in “Dagsavisen” [96]. She warns that CPs based on different standardized package solutions make user participation more difficult as the patient only gets to choose from the treatment involved in the CP. She fears that CPs will move mental health services in the opposite direction because of the standardized approach. “*It would be nice, after all the talk about the importance of user involvement, if knowledge from experience, help on the premises of the patient, *etc*., finally started to show up in real life”.*

Keeping an individualized perspective while at the same time standardizing elements meet some challenges, as is expressed in the above quotation. However, the rhetoric that this is indeed possible exist in the political domain and is further expressed by Prime Minister Erna Solberg in a speech at a meeting at the National Center for Experience-based Competence in 2015 [97]. In her speech, she talks about an increased focus on user-driven mental health services and medication-free services for patients to choose from. She states:

*“We have to stop asking the patients: what is wrong with you? We have to start asking the patient: What is important to you? Listening to the patients also means listening to those who want medical-free services*”.

Despite the promise that the CP will take the patient’s wishes seriously, she also, in the same speech, claims:

*“Clinical pathways will ensure that the services provided are based on the best evidence-based practice for the disease.… This involves clear deadlines for the different steps in the treatment.…This will give more equal treatment despite geography and different institutions”.*

The ideas of standardization and individualization clearly collide between the different groups of actors’ logics, and interpretations of what they mean and how they materialize as practices within mental health care. The final result was transformed into something quite different than the pathway around which the public discussion circulated. Understanding this process means examining the work of those designating the pathway “Mental illness, adults”. However, this process is influenced by the values and institutional logics of the different groups, as is shown when analysing the work process leading up to the final product. A complete overview of each group’s institutional logic and their relations to CPs is found in Table 2.

It is obvious when looking at the table that the two actors relate to pathways differently and that their sense making is guided by already established values, assumptions and rules to be found within their professional identity. How each logic has influenced the final product and the making of the pathway is elaborated in the next, and final, part of the analysis.

### Organizing as negotiation: the directorate of health as a mediator

#### The conferences

Thornton and Ocasio [70] remind us that actors in the field are strongly guided by values in their way of viewing their world and in their organizing of time and space. When taking this into consideration, the process of making a pathway means creating an arena to which the different groups bring their own institutional values and ideas. Furthermore, the work groups could be viewed as an informal negotiation arena in which the logics of professionals and politicians meet. The actors’ acceptance of these logics creates leverage in the informal “negotiation”. When pursuing an understanding of the making of the pathway, the issue of identification is of vital importance. In social situations encountered by the actors in the field, they activate a wide variety of social identities from different institutional logics [78]. Actors who work in the Directorate of Health are operating in the field between health professionals executing their daily work and politicians deciding on different health care policies and strategies. Identification is therefore based on different institutional logics. Furthermore, this gives them unique knowledge of both institutional logics. This sense making enables them to know which elements are negotiable and which are not.

“*When we first got the assignment from the ministry, we thought, Well, if clinical pathway is an answer to a question, then what is that question?*”

The following quotation from a health professional working in the Directorate of Health shows the start of a “muddling through” [24], the process where negotiation occurs and where the different logics, values, and assumptions clash, affecting the making of and final result of the pathway. Furthermore, it shows how the Government wanted to transfer the idea from somatic health in a new area without thorough knowlegde of the mental health care field. Shortly after the Directorate of Health was given the assignment by the ministry, conferences with different professionals working within mental health services were arranged. The agenda and motivation behind the meetings were to provide a space where ideas concerning organization of mental health services, and pathways were to be discussed. Based on mental health services’ heterogeneity, these conferences often led to heavy discussions and disagreements on how to provide the right kind of treatment. However, this did not happen at the conferences where the content of the pathways, and organization of mental health services were to be discussed. This is what one user group representative recruited to the work group had to say about the themes that were discussed at the conference:

“*People at the conferences were completely agreeing. That was something I found interesting because normally there are big disagreements. The participants all repeated the same message: ‘We cannot have diagnosis-specific treatment as they have in Denmark.…’ Before it was decided what kind of pathway we should make, we found out that we had to bend the order from the department. The order from the department said that the pathways would be organized around diagnosis. We bent it by putting several diagnoses into the same pathway”.*

The above quotation indicates how guiding health professional values such as discretion and autonomy are within this professional logic. *It is expected that institutional logics affect organizational decision making by steering the attention of decision makers* [98]. This attention was steered towards a common goal where the focus centred around protecting professionals’ values and boundaries. The conference could have been an arena in which the different actors actively involved themselves in discussions regarding the issues mental health care services face today, and different solutions applicable. However, as the informant stated above, this did not happen. The protection of values is aligned with Friedland’s [71] research into how values are the foundation of the ontology and something that guides overall sense making [71]. An explanation for the phenomenon could be that withholding values of discretion and autonomy is more important because the clinical pathway is experienced as a threat to their work. This made the discussion around the CP circulate around the themes of CPs and their inappropriateness in mental health services.

#### The work group

The work group also appeared to function without much of the disagreement that normally occurs when different actors in a heterogeneous field come together. This is explained by a psychiatrist in the work group:

“*The work group functioned really well. Everyone was heard, and there were no big conflicts. Not that we just sat and played along, but there were no big contentious issues. There was not anything to be discussed that the members were really disagreeing on*”.

Another health professional in the work group said:

“*Everyone was heard; everyone was listened to. Nothing like what is happening in meetings in (mental health services red.) real life”.*

This shows the unfolding of the subtle negotiation process, and a possible interpretation is that the Directorate of Health had already made sure to rule out disagreements by organizing the pathways in such a manner that discretion and autonomy were withheld, showing that part of the negotiations where the importance of retaining each logic’s values was handled by the directorate. Considering this matter, a psychiatrist in the work group answered as follows when asked how much the members of the work group were able to influence the making of the pathway:

“*We had received a template that was a bit like the cancer pathways. Like what titles to fill in, and we were made clear from our first meeting that this was only logistics, and this was repeated through the process. And I think many were surprised about that. We started out thinking that we were there to recommend what diagnostic tools to use, but we were wrong. We were not allowed to recommend anything concrete”*.

Despite accommodating health professionals’ values, it was well known to the members of the work group that this was part of a negotiation process and that not all of their needs would be accommodated by the Directorate of Health.

“*The government wanted something in return. I understood that immediately. And then they need something to evaluate; there must be some codes involved. And we must remember the coding, and that is the challenge. I really do not understand how we are going to make it work”.*

The coding and the extra work related to it was an overall concern as well as a cause of general discontent among most of the professionals in the work group, and the above quotation from a psychologist indicates in stating that “they wanted something in return” that this was a negotiation between the two logics. Furthermore, it shows that the needs of the political logic for rationalization and control are well known to the professional logic. In addition, the need for control of health professionals’ work is expressed as a burden for the actors involved, and the frustration over this is clearly expressed in the above quotation. Pursuing this from the perspectives of different values, the quotation also indicates the polarized view on the values behind a CP. Control for a political logic means efficiency and better services for patients. Control for the health professionals’ logic means losing time that could be spent on patient treatment and instead using it on administrative tasks. However, the codes involved do not directly interfere with discretion, meaning that individuality and flexibility in terms of treatment are not lost, so their core values are still intact.

One of the main issues regarding CPs, from early on and to date, is the term and the issues associated with the idea of a CP. A CP is a way of organizing services within somatic medicine, and cancer treatment is one of them. Moreover, the elements of standardized work practices and EBM provoke actors in the professional logic, as they collide with the core values of discretion and autonomy. However, for the political logic, the name CP indicates success, as it has been proven to reduce waiting time and unwanted variation and is more or less portrayed as an achievement [99]. The name “clinical pathway” legitimizes political will and action in the field of health care services. The name is also misleading because the clinical aspect of the pathway was lost early in its making. Some of the critique from actors in the field of mental health services could possibly have been avoided by naming the pathways differently and leaving out the negative connotations that these actors associate with the name. However misleading the name was, it was not for sale:

“*It was not our call to decide the name of the clinical pathways. It was given. So… there has been quite a lot of resistance to it. We addressed this with the ministry. The ministry is familiar with these issues, and they have been for some time. They kept the name ‘clinical pathway’; it was not our call to make”.*

The quotation from a health professional within the Directorate of Health also indicates the possibility that the department knew “how to choose their battles”. The name was not something to fight for, as this did not interfere with the issues in the conflict regarding CP suitability in mental health services. The name, however, caused much unwanted noise, as its connotations caused the professionals to feel threatened long after their autonomy and discretion were safe.

#### The final product: from CP to logistic pathway

**Fig. 1:**
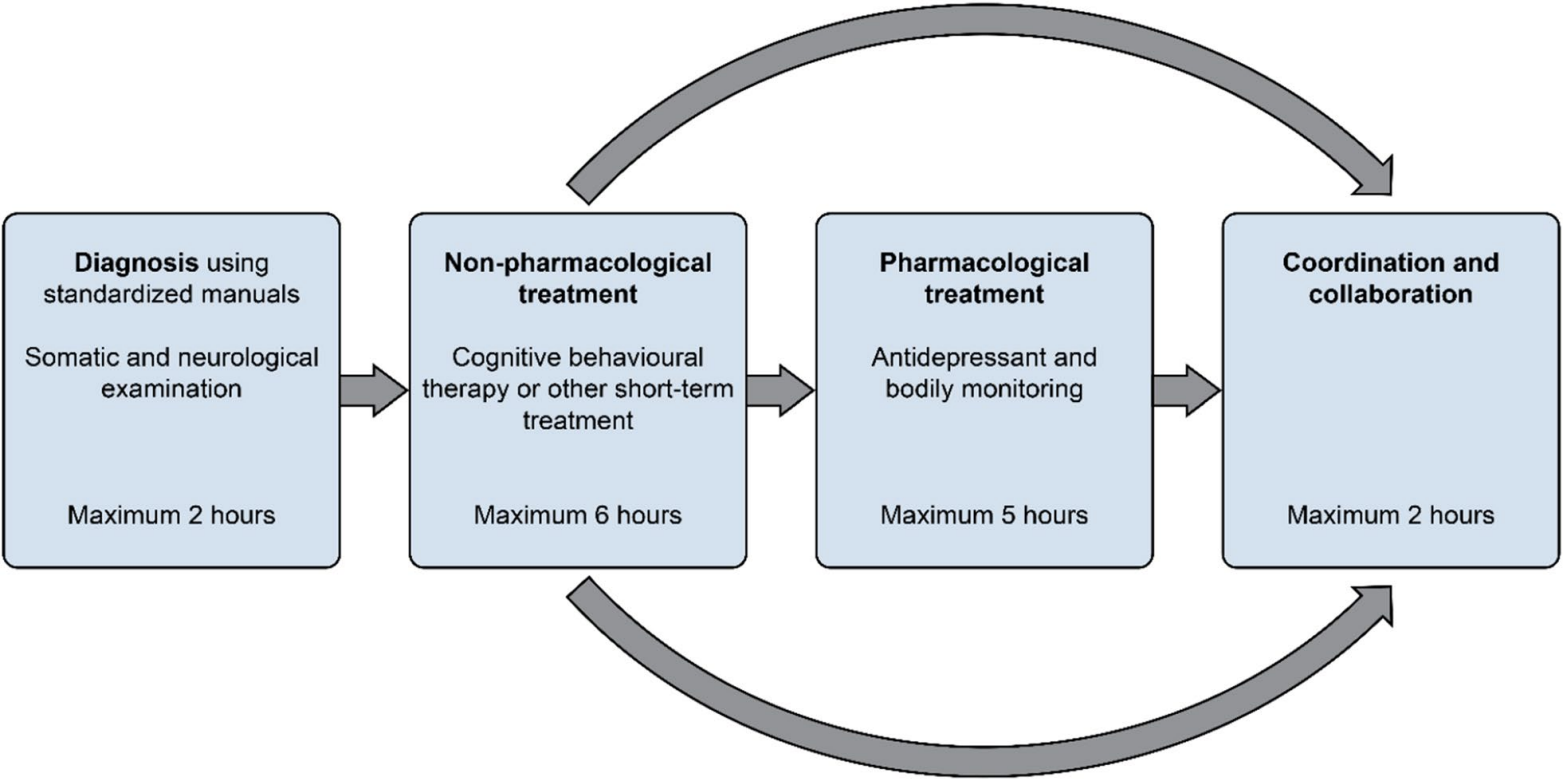
“CP for depression, Denmark”

At the time of implementation, the number of pathways had been reduced from 22 to 9. As the idea of having diagnosis-specific CPs based on EBM with standardized practices copied from the Danish model, such as Fig. 1*“CP for depression, Denmark* “ illustrates, the outcome had moved far off the original intention.

**Fig. 2:**
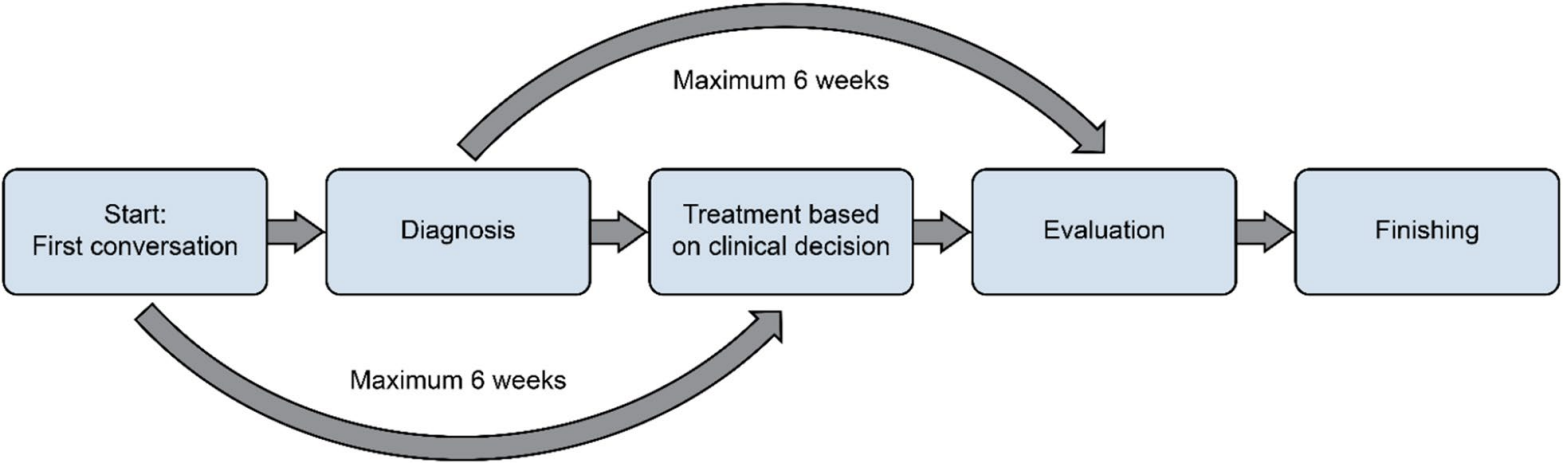
“Patient pathway, mental illness adults”

The final result of the “Pathway for mental illness, adults” is utitlized to treat depression, and became a general pathway involving several groups of patients. Within this pathway, all patients belonging to the same service area are generalized, making the pathway a description of the services. The pathway explains the process from diagnosis through treatment and finalizing treatment. The different steps involve time frames that need to be coded, but without stating which diagnostic tools should be used, nor does the pathway explain preferred treatment strategies for the different diagnoses. Figure 2 “*Patient pathway, mental illness adults*” illustrates the general pathway for treatment for adults.

Finally, the pathway is guided by five overall goals: 1. increased user participation and satisfaction; 2. coherent and coordinated patient pathways; 3. avoidance of unneccessary waiting time for diagnostication, treatment and follow-up; 4. more equal services despite geographical location; and 5. improved focus on somatic health and lifestyle. The overall goals were meant to be guidelines for the implementation strategies. Furthermore, the pathways change professional work practices by imposing a more rigid system of documentation and coding of the different steps, involving a more bureaucratic system. This documentation makes the time spent by health professionals per patient in their daily work more transparent, and enables it to be monitored by the political level, but without touching professional discretion and autonomy. The pathway in the matter of the previous discussion therefore ends up being a product negotiated from the values presented in the institutional logics.

## Discussion

The analytical discussion also shows the downside of having sense making guided by strong values associated with a specific institutional logic. It seems to be an almost taken-for-granted way of viewing how a certain health care service should be organized without questioning whether this is, in fact, the best solution. Those at the political level assumed that transferring successful ideas from other hospitalization services could be easily done, but without having a thorough knowledge of the field. They did not adopt a context-sensitive focus on understanding the nature of the problems and how they might be solved, which is considered a condition for appropriate problem solving [100, 101]. Therefore, a thorough understanding of the field and the mission is essential for every decision maker’s competence. In the case of mental health services, this involves understanding empathy for patients and health professionals’ work, respect for professional knowledge, responsibility for limited economic resources and social trust [100].

Furthermore, although health professionals guided by their values, namely, discretion and autonomy, have a thorough knowledge of the field and its weaknesses, it seems that retaining these values is sometimes more important than actively involving themselves in the debate regarding the negative aspects and issues of the current organization of mental health services. According to Argyris [101], in Falkenstrøm et al. [102], how a certain problem is defined and solved may be the cause of the problem. Therefore, it is necessary to question the underlying assumptions and principles and seek a broader, more dynamic, and critical understanding of the problem. This way of learning and viewing things differently implies a change in the mental model that forms the basis for decision making [101]. Nevertheless, a review of theory on institutional logics shows that a change in a mental model means opposing values forming strong identities, and in the quest for a new perspective and understanding, one could possibly end up losing one’s professional identity.

Conclusively, CPs are understood in a polarized terminology by health professionals, where being concerned with efficiency means not caring for patients, and the public discussion regarding mental health care became a battlefield where their main motivation was to discredit the idea of the CP and its suitability in mental health services instead of engaging in what could possibly have become a more constructive discussion.

## Conclusions

This article provides a thorough examination of the making of a new health reform in Norwegian mental health services: the idea of a CP in Norwegian mental health services from 2015. This article sheds light on some of the issues that occur in the making of new health reforms. In the health care field, different actors interpret the ideas of the CP differently, bringing the values and assumptions associated with their institutional logics to this understanding. In the ensuing alternation, a negotiation process occurs where the guiding values decide what elements that are up for negotiation. Within the professional logic, the values of autonomy and discretion are not for sale, and this is accepted by the political logic because they can retain their values of control and efficiency. The Norwegian Directorate of Health led the way in the process. Actors who work there have a health professional background but work within the political field, giving them access to both logics. This knowledge of the values made the process a rather seamless negotiation, as both logics were able to retain the core of their identity.

## Data Availability

Not applicable.

## Abbreviations

CP: Clinical pathway
EBM: Evidence based medicine
NPM: New public management

## Footnotes

1 The Norwegian Directorate of health, official statistics, mental health care.
