# Correction to: Organizing as negotiation: the construction of a pathway in Norwegian mental health services

**DOI:** 10.1186/s13033-021-00498-4

**Published:** 2021-09-25

**Authors:** Tine Nesbø Tørseth

**Affiliations:** 1 The Mohn Centre for Innovation and Regional Development, Western Norway University of Applied Sciences, is a Research and Competence Centre within the Field of Responsible Innovation, Bergen, Norway; 2 The University of Bergen, Bergen, Norway

## Correction to: Int J Ment Health Syst (2021) 15:26 https://doi.org/10.1186/s13033-021-00451-5

In the original version [1] of this article the author name, Tine Nesbø Tørseth was incorrectly written as Tine Nesboe Toerseth.

The original article has been corrected.
